# Cremation in Denmark

**Published:** 1881-10

**Authors:** 


### Cremation in Denmark.

A new impulse to cremation has recently been given by the organization and work of the society in Denmark. At a meeting held in Copenhagen, May 31, it was shown that the society counted fourteen hundred and nine members, among whom were eighty-three physicians and some prominent clergymen. Opposition and prejudice have to a large extent died away, even among clergymen. In the furnace projected by the Danish society corpses are to be reduced to ashes in a little over one hour, and it is calculated that the cost of incineration will be reduced to the insignificant sum of from three to five crowns—between one and two dollars. This economical feature of the project has met with great favor among the poorer classes, who complain that it is expensive to live, but much more expensive to die in the Danish capital.

